# ﻿Bottlebrush and Myrtle twig canker caused by *Neopestalotiopsis* species: an emerging canker-causing group of fungi in Italy

**DOI:** 10.3897/mycokeys.106.121520

**Published:** 2024-06-21

**Authors:** Dalia Aiello, Giorgio Gusella, Giuseppa Rosaria Leonardi, Giancarlo Polizzi, Hermann Voglmayr

**Affiliations:** 1 Dipartimento di Agricoltura, Alimentazione e Ambiente, sez. Patologia Vegetale, University of Catania, Via S. Sofia 100, 95123 Catania, Italy University of Catania Catania Italy; 2 Department of Botany and Biodiversity Research, University of Vienna, Rennweg 14, 1030 Vienna, Austria University of Vienna Vienna Austria

**Keywords:** Bottlebrush, canker, myrtle, *Neopestalotiopsis*, phylogeny

## Abstract

Pestalotioid fungi were isolated in pure culture from symptomatic plants of *Callistemonlaevis*, *C.viminalis*, *Lumaapiculata* (marketed as “*Myrtusluma*”), Myrtuscommunissubsp.tarentina, and M.communisvar.microphylla (*M.communis* ’Microphylla’), showing twig canker, dieback and defoliation. The isolates were identified to species by ITS, *tef1* and *tub2* sequences, which revealed the presence of six species of *Neopestalotiopsis* (*N.camelliae-oleiferae*, *N.hispanica*, *N.iberica*, *N.rosae*, *N.rosicola*, and *N.zakeelii*) and one species of *Pestalotiopsis* (*P.biciliata*). While most species were isolated only once or twice, the majority of isolates belonged to *N.rosae* (13) and *N.hispanica* (8). Pathogenicity was investigated by pathogenicity tests on all hosts, which confirmed the pathogenicity of all *Neopestalotiopsis* species on at least some of the hosts tested, while *P.biciliata* did not cause any disease symptoms. *Neopestalotiopsishispanica* and *N.rosae* caused symptoms in all hosts of the present study, while the other *Neopestalotiopsis* species tested showed no symptoms on *Lumaapiculata*.

## ﻿Introduction

The Bottlebrush (*Callistemoncitrinus* (Curtis.) Skeels.) and myrtle (*Myrtuscommunis* L.) are evergreen shrubs belonging to Myrtaceae. Bottlebrushes refer to *Callistemon* species, a group of plants native to Australia. In recent years, Australian plants, especially *Callistemon* species, have aroused interest in Mediterranean countries as ornamental plants mainly due to their aesthetic value and great adaptability to Mediterranean environmental conditions (Lippi et al. 2003).

Little is known about fungal diseases of bottlebrush. Some species of *Calonectria* (*Ca.cylindrospora*, *Ca.mexicana*, *Ca.pauciramosa*, and *Ca.pseudomexicana*) were reported causing leaf spot, blight, stem lesion and damping-off on different accessions of bottlebrushes in Europe (Pérez-Sierra et al. 2007; Polizzi et al. 2007; Vitale et al. 2013; Aiello et al. 2022) and the African continent (Lombard et al. 2011). Other causal agents of leaf spot have been reported on *C.citrinus*, including species belonging to the genera *Alternaria*, *Botryosphaeria*, *Cercospora*, *Colletotrichum*, *Phyllosticta*, and *Selenophoma* (Alfieri et al. 1984; Pennycook et al. 1989; Gadgil 2005). Recently, in Iran the new species *Xenodidymellairanica* (Didymellaceae) was isolated and described from leaf spot and blight of *C.citrinus* (Ahmadpour et al. 2022). In addition, stem canker was reportedly caused by species of *Leptosphaeria* and *Phomopsis*, stem gall by *Cophinformatumefaciens* (syn. *Sphaeropsistumefaciens*), and root rot diseases by *Rhizoctoniasolani* and species of *Phytophthora* and *Pythium* (Alfieri et al. 1984; Shivas 1989; Erwin and Ribeiro 1996; Cacciola et al. 2009).

Myrtle is widely distributed in the Mediterranean area forming spontaneous bushes, and in Italy is particularly occurring in coastal areas and islands. Two subspecies are currently accepted within this species, Myrtuscommunissubsp.communisandsubsp.tarentina (Medda and Mulas 2021; POWO 2024). Myrtle is cultivated for many different purposes such as pharmaceutical, cosmetic, ornamental (especially due to high vegetative vigor, the bright green color of the leaves, and the abundant multi-colored flowering), and food industries (Mulas et al. 1998; Medda and Mulas 2021). Although myrtle is investigated worldwide under many different aspects due to its manifold uses, few data are available regarding diseases affecting this crop. Amongst fungal diseases, leaf spot and crown and root disease caused by *Ca.mexicana*, *Ca.pauciramosa*, and *Ca.pseudomexicana* have been reported in Europe (Polizzi and Crous 1999; Henricot and Beales 2003) as well as in the United States (Koike and Crous 2001) and in Tunisia (Lombard et al. 2011).

In addition, the myrtle rust disease caused by *Austropucciniapsidii* (syn. *Pucciniapsidii*) (Uredinales, Pucciniaceae) is considered an important quarantine threat (Glen et al. 2007; Fensham et al. 2020), which became notorious when it started to infect various species of the Myrtaceae (Coutinho et al. 1998; Glen et al. 2007; Carnegie et al. 2010; Morin et al. 2012; Roux et al. 2013). Recently, Italian provenances of *M.communis* were shown to be highly susceptible to *Austropucciniapsidii*, highlighting a significant threat to myrtle cultivation if the pathogen were accidentally introduced to the Mediterranean (Paap et al. 2023).

In Sicily (southern Italy), recent surveys of a nursery revealed the presence of many myrtle and bottlebrush plants affected by twig cankers and dieback. Investigations conducted in recent years in Sicily, in greenhouses and also in the field, showed an increase of these symptoms in many different crops, especially ornamental plants, caused mainly by Botryosphaeriaceae spp. (Aiello et al. 2020; Gusella et al. 2021; Costanzo et al. 2022; Fiorenza et al. 2022a). Canker-causing pathogens are under investigation worldwide for many different aspects concerning the complex etiology of the diseases, their epidemiology, wide host range and difficulties in management (Guarnaccia et al. 2022). Among the canker-causing pathogens, pestalotioid fungi have been increasingly investigated in recent years (Silva et al. 2020; Diogo et al. 2021; Fiorenza et al. 2022b; Li et al. 2022; Santos et al. 2022; Zheng et al. 2023). Maharachchikumbura et al. (2014) revised *Pestalotiopsissensu lato* dividing it into three distinct genera, *viz. Pestalotiopsis*, *Pseudopestalotiopsis*, and *Neopestalotiopsis*. These fungi are widely distributed in tropical and temperate areas and are frequently encountered as endophytes and plant pathogens causing stem-end rot, stem and leaf blight, trunk disease, and cankers (Maharachchikumbura et al. 2014). In Sicily, recent studies of young avocado trees showing canker and wood discoloration led to the identification of two *Neopestalotiopsis* species responsible for those symptoms, namely *N.rosae* and *N.siciliana* sp. nov. (Fiorenza et al. 2022b). Moreover, Fiorenza et al. (2022b) noticed frequent necroses and cankers at the grafting point, reinforcing the assumption that the propagation processes are crucial for pestalotioid infections (Espinoza et al. 2008). Therefore, myrtle and bottlebrush plants showing symptoms of twig canker and dieback were investigated in the present study to provide a deeper insight into etiology. The aims of this study were to: ii) identify and characterize the etiological agents; ii) test their pathogenicity.

## ﻿Materials and methods

### ﻿Survey and fungal isolations

The survey was conducted from September 2021 in a nursery in Catania (eastern Sicily). Symptomatic plants of *Callistemonlaevis*, *C.viminalis*, *Lumaapiculata* (marketed as “*Myrtusluma*”), Myrtuscommunissubsp.tarentina, and M.communisvar.microphylla (*M.communis* ’Microphylla’), showing twig canker, dieback and defoliation were brought to the laboratory of the Dipartimento di Agricoltura, Alimentazione e Ambiente for further analyses. Specifically, small pieces of diseased wood tissue from cankered twigs were surface sterilized for 1 min in 1.5% sodium hypochlorite (NaOCl), rinsed in sterile water, dried on sterile absorbent paper in a laminar flow hood and then placed on potato dextrose agar (PDA, Lickson, Italy) amended with 100 mg/L of streptomycin sulfate (Sigma-Aldrich, MO, USA) to prevent bacterial growth. The Petri plates were then incubated at 25 °C for 7 days until the fungal colonies were large enough to be examined. For M.communissubsp.tarentina and *M.communis* ’Microphylla’, isolations were also conducted from the leaves as described above. Colonies of interest were transferred onto fresh PDA plates and then single hypha isolates were obtained from pure cultures. Fungal isolates were stored as mycelial plugs in sterile water in the fungal collection of the laboratory with the strain identifiers ML (isolates from *Lumaapiculata*), MT (isolates from M.communissubsp.tarentina), MP (isolates from *M.communis* ’Microphylla’) or CV (isolates from *Callistemon*).

### ﻿DNA extraction, PCR and sequencing

A total of 26 representative isolates from *Callistemon* spp., as well as from *Luma* and *Myrtus*, were chosen for molecular and phylogenetic analysis. DNA was extracted from single hypha isolates grown on PDA. The mycelium was scraped off and processed following manufacturer’s instructions of the Wizard Genomic DNA Purification Kit® (Promega Corporation, WI, USA).

The following loci were amplified and sequenced: the terminal 3’ end of the small subunit nuclear ribosomal DNA (nSSU rDNA) and the complete internal transcribed spacer region (ITS1-5.8S-ITS2) of the rDNA region with primers V9G (de Hoog and Gerrits van den Ende 1998) and ITS4 (White et al. 1990); a ca 0.5 kb fragment of the translation elongation factor 1-alpha (*tef1*) gene with primers EF1-728F (Carbone and Kohn 1999) and TEFD_iR (Voglmayr et al. 2018); and a ca. 0.95 kb fragment of the beta tubulin (*tub2*) gene with primers T1D (Voglmayr et al. 2019) and BtHV2r (Voglmayr et al. 2016, 2017). PCR products were purified using enzymatic PCR cleanup (Werle et al. 1994) as described in Voglmayr and Jaklitsch (2008). DNA was cycle-sequenced using the ABI PRISM Big Dye Terminator Cycle Sequencing Ready Reaction Kit v. 3.1 (Applied Biosystems, Warrington, U.K.) with the same primers as in PCR. Sequencing was performed on an automated DNA sequencer (3730xl Genetic Analyzer, Applied Biosystems). The resulting DNA sequences were assembled with Lasergene SeqMan Pro (DNASTAR, Madison, WI, USA). The sequences generated during the present study were deposited in Genbank (Table 1).

**Table 1.** Symptomatology observed on different plant species investigated in this study.

Plant species	Symptoms	Disease incidence (%)
*Callistemonlaevis*	small twig canker; dieback and defoliation	30
*C.viminalis*	small twig canker; dieback and defoliation	30
*Lumaapiculata*	small twig canker, dieback, and low defoliation	20
*Myrtuscommunis* ‘Microphylla’	small twig canker, dieback, and very low defoliation	20
M.communissubsp.tarentina	small twig canker, dieback, and high defoliation	70

### ﻿Analysis of sequence data

For the phylogenetic analysis, a combined matrix of ITS rDNA, *tef1* and *tub2* sequences was produced. The newly generated sequences were aligned to a representative matrix of GenBank sequences of *Neopestalotiopsis* and *Pestalotiopsis*. For *Neopestalotiopsis*, all 88 described species for which sequences were available were included in the matrix, preferentially with ex-type sequences. Sequences of the four taxa of *Pestalotiopsis* which were the closest match to the single *Pestalotiopsis* isolate of the current study were added as outgroup. The GenBank accession numbers of the sequences used in this analysis are given in Table 1.

All alignments were produced with the server version of MAFFT (www.ebi.ac.uk/Tools/mafft), checked and refined using BioEdit version 7.2.6 (Hall 1999). For some sequences, highly deviating leading or trailing sequence regions, which were likely affected by sequencing errors, were removed from the alignment, as well as primer residue sequences that were present in some GenBank sequences. The ITS rDNA, *tef1* and *tub2* matrices were combined for subsequent phylogenetic analysis.

Maximum likelihood (ML) analyses were performed with RAxML (Stamatakis 2006) as implemented in raxmlGUI 2.0 (Silvestro and Michalak 2012), using the ML + rapid bootstrap setting and the GTRGAMMA+I substitution model (selected as the best model by Modeltest) with 1000 bootstrap replicates. The matrix was partitioned for different gene regions. For evaluation and discussion of bootstrap support, values below 70% were considered low, between 70 and 90% medium/moderate, above 90% high and 100% maximum.

Maximum parsimony (MP) bootstrap analyses were performed with PAUP v. 4.0a169 (Swofford 2002), with 1 000 bootstrap replicates using five rounds of heuristic search replicates with random addition of sequences and subsequent TBR branch swapping (MULTREES option in effect, steepest descent option not in effect, COLLAPSE command set to MINBRLEN, each replicate limited to 1 million rearrangements) during each bootstrap replicate. All molecular characters were unordered and given equal weight; analyses were performed with gaps treated as missing data; the COLLAPSE command was set to minbrlen.

### ﻿Morphological investigation

Morphology of conidia was checked from sporulated cultures on PDA, CMD (CMA: Sigma, St Louis, Missouri; supplemented with 2% (w/v) D(+)-glucose-monohydrate) or 2% MEA (2% w/v malt extract, 2% w/v agar-agar; Merck, Darmstadt, Germany) plates grown at room temperature at ambient light. Conidia, prepared in tap water on microscope slides, were investigated in a Zeiss Axio Imager. A1 compound microscope.

### ﻿Pathogenicity test

To determine the ability of the fungal species to infect and induce symptoms, seven representative isolates were selected for pathogenicity tests: *Neopestalotiopsiscamelliae-oleiferae* ML3, *N.hispanica* ML8, *N.iberica* MP13, *N.rosae* MT32, *N.rosicola* MP29, *N.zakeelii* MP30, and *P.biciliata* MP11 were inoculated on potted plants of *Callistemonlaevis*, *C.viminalis*, M.communissubsp.tarentina, *M.communis* ‘Mycrophylla’, and *L.apiculata*. Each fungal species was inoculated on three plants (replicate) of each plant species. For each plant, six twigs were inoculated as sub-replicates. The inoculum consisted of a small piece (~ 0.3–0.5 mm^2^) of mycelial plug from 15-day-old cultures on PDA. The bark was first gently scraped using a sterile blade and then the mycelial plug was inserted upside down onto the wound. The wounds were sealed with Parafilm to prevent desiccation. Controls consisted of sterile PDA. All inoculated plants were moved to a growth chamber with a 12 h photoperiod and maintained at 25 ± 1 °C. The plants were regularly watered and monitored weekly for development of symptoms. Final symptoms evaluation was conducted 30 days after inoculations. Re-isolations were performed as described above to fulfill Koch’s postulates.

## ﻿Results

### ﻿Survey and fungal isolations

**Figure 1.**
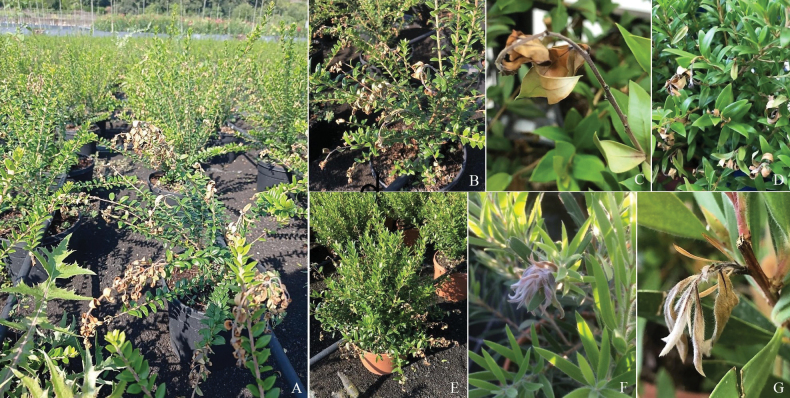
Disease symptoms caused by *Neopestalotiopsis* spp. in nursery: blight and defoliation on Myrtuscommunissubsp.tarentina**A–B***Lumaapiculata***C–D***M.communis* ‘Microphylla’ **E***Callistemonviminalis***F** and *C.laevis***G**.

Symptoms observed in the nursery included twig canker, dieback, internal wood necrosis and defoliation (Fig. 1). Symptomatology details and disease incidence for each plant species are described in Table 1. Fungal isolations constantly yielded *Neopestalotiopsis*-like colonies. A total of 56 *Neopestalotiopsis*-like isolates were stored in the fungal collection.

### ﻿Phylogenetic analysis and species identification

**Figure 2.**
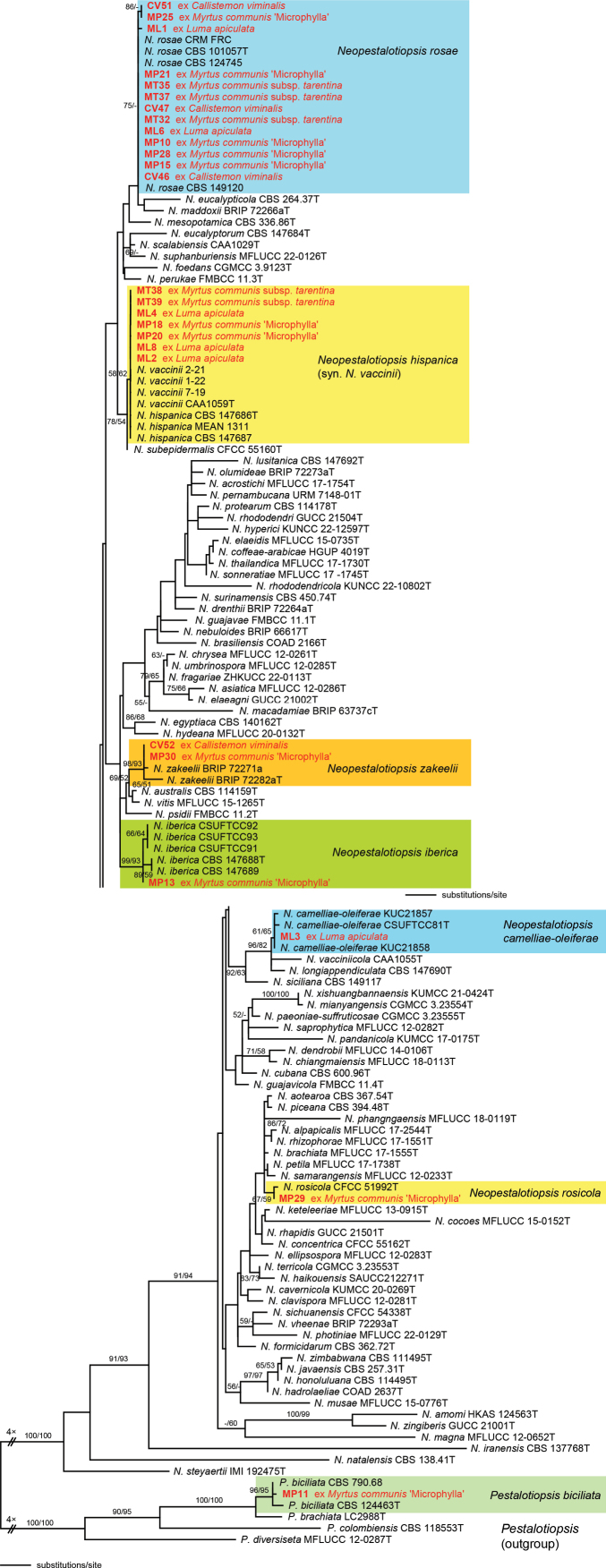
Phylogram of the best ML tree (-lnL = 10329.8215) revealed by RAxML from an analysis of the combined ITS-*tef1*-*tub2* matrix of *Neopestalotiopsis*, showing the phylogenetic position of the isolates obtained from diseased Myrtacae. Strains marked by an “T” at the end of the strain designation represent ex-type strains. ML and MP bootstrap support above 50% are given above or below the branches. The broken branches were scaled to one-quarter.

The combined multilocus matrix used for phylogenetic analyses comprised 2458 characters (539 from the ITS, 978 from *tef1*, and 941 from *tub2*), of which 414 were parsimony informative (66 from ITS, 170 from *tef1*, and 178 from *tub2*). The best ML tree (-lnL = 10329.8215) obtained by RAxML is shown in Fig. 2. While the genus *Neopestalotiopsis* and a few deeper nodes were highly supported, most of the tree backbone received little or no bootstrap support. However, the isolates of the present study could be assigned to species level, based on the results of the phylogenetic analyses (Fig. 2), viz. one species of *Pestalotiopsis* and 6 species of *Neopestalotiopsis*: isolate MP11 was revealed as *Pestalotiopsisbiciliata*, isolate MP29 as *Neopestalotiopsisrosicola*, ML3 as *N.camelliae-oleiferae*, MP13 as *N.iberica*, and the two isolates CV52 and MP30 as *N.zakeelii*. However, the majority of isolates belonged to two species, viz. eight isolates to *N.hispanica* (syn. *N.vaccinii*) and 13 to *N.rosae* (see Table 2 and Fig. 2).

**Table 2.** Isolates used in the molecular analyses in this study.

Species	Strain^1^	Host/Substrate	Origin	GenBank Accession Numbers^2^		
				ITS	*tef1*	*tub2*
*Neopestalotiopsisacrostichi*	MFLUCC 17-1754^T^	*Acrostichumaureum*	Thailand	MK764272	MK764316	MK764338
*N.alpapicalis*	MFLUCC 17-2544^T^	*Rhizophoramucronata*	Thailand	MK357772	MK463547	MK463545
*N.amomi*	HKAS 124563^T^	*Amomumvillosum*	China	OP498012	OP653489	OP752133
*N.aotearoa*	CBS 367.54^T^	Canvas	New Zealand	KM199369	KM199526	KM199454
*N.asiatica*	MFLUCC 12-0286^T^	Unidentified tree	China	JX398983	JX399049	JX399018
*N.australis*	CBS 114159^T^	*Telopea* sp.	Australia	KM199348	KM199537	KM199432
*N.brachiata*	MFLUCC 17-1555^T^	*Rhizophoraapiculata*	Thailand	MK764274	MK764318	MK764340
*N.brasiliensis*	COAD 2166^T^	*Psidiumguajava*	Brazil	MG686469	MG692402	MG692400
*N.camelliae-oleiferae*	CSUFTCC81^T^	*Camelliaoleifera*	China	OK493585	OK507955	OK562360
*N.camelliae-oleiferae*	KUC21857	*Phyllostachysbambusoides*	South Korea	OR654966	OR693485	OR693494
*N.camelliae-oleiferae*	KUC21858	*Phyllostachysbambusoides*	South Korea	OR654967	OR693486	OR693495
*N.camelliae-oleiferae*	**ML3**	*Lumaapiculata*	**Italy**	**PP146586**	**PP174965**	**PP197178**
*N.cavernicola*	KUMCC 20-0269^T^	Cave rock surface	China	MW545802	MW550735	MW557596
*N.chiangmaiensis*	MFLUCC 18-0113^T^	Dead leaves	Thailand	N/A	MH388404	MH412725
*N.chrysea*	MFLUCC 12-0261^T^	*Pandanus* sp.	China	JX398985	JX399051	JX399020
*N.clavispora*	MFLUCC 12-0281^T^	*Magnolia* sp.	China	JX398979	JX399045	JX399014
*N.cocoes*	MFLUCC 15-0152^T^	*Cocosnucifera*	Thailand	NR_156312	KX789689	N/A
*N.concentrica*	CFCC 55162^T^	*Rosarugosa*	China	OK560707	OM622433	OM117698
*N.coffeae-arabicae*	HGUP 4019^T^	*Coffeaarabica*	China	KF412649	KF412646	KF412643
*N.cubana*	CBS 600.96^T^	Leaf litter	Cuba	KM199347	KM199521	KM199438
*N.dendrobii*	MFLUCC 14-0106^T^	*Dendrobiumcariniferum*	Thailand	MK993571	MK975829	MK975835
*N.drenthii*	BRIP 72264a^T^	*Macadamiaintegrifolia*	Australia	MZ303787	MZ344172	MZ312680
*N.egyptiaca*	CBS 140162^T^	*Mangiferaindica*	Egypt	KP943747	KP943748	KP943746
*N.elaeagni*	GUCC 21002^T^	*Elaeagnuspungens*	China	MW930716	MZ203452	MZ683391
*N.elaeidis*	MFLUCC 15-0735^T^	*Elaeisguineensis*	Thailand	ON650690	ON734012	N/A
*N.ellipsospora*	MFLUCC 12-0283^T^	Dead plant materials	China	JX398980	JX399047	JX399016
*N.eucalypticola*	CBS 264.37^T^	*Eucalyptusglobulus*	N/A	KM199376	KM199551	KM199431
*N.eucalyptorum*	CBS 147684^T^	*Eucalyptusglobulus*	Portugal	MW794108	MW805397	MW802841
*N.foedans*	CGMCC 3.9123^T^	Mangrove plant	China	JX398987	JX399053	JX399022
*N.formicidarum*	CBS 362.72^T^	Dead *Formicidae* (ant)	Ghana	KM199358	KM199517	KM199455
*N.fragariae*	ZHKUCC 22-0113^T^	Fragaria×ananassa	China	ON553410	ON569076	ON569075
*N.guajavae*	FMBCC 11.1^T^	*Psidiumguajava*	Pakistan	MF783085	MH460868	MH460871
*N.guajavicola*	FMBCC 11.4^T^	*Psidiumguajava*	Pakistan	MH209245	MH460870	MH460873
*N.hadrolaeliae*	COAD 2637^T^	*Hadrolaeliajongheana*	Brazil	MK454709	MK465122	MK465120
*N.haikouensis*	SAUCC212271^T^	*Ilexchinensis*	China	OK087294	OK104877	OK104870
*N.hispanica*	CBS 147686^T^	*Eucalyptusglobulus*	Portugal	MW794107	MW805399	MW802840
*N.hispanica*	CBS 147687	*Eucalyptusglobulus*	Spain	MW794113	MW805401	MW802846
*N.hispanica*	MEAN 1311	*Eucalyptusglobulus*	Portugal	MW794106	MW805400	MW802839
*N.hispanica*	**ML2**	*Lumaapiculata*	**Italy**	**PP146593**	**PP174983**	**PP197171**
*N.hispanica*	**ML4**	*Lumaapiculata*	**Italy**	**PP146594**	**PP174984**	**PP197172**
*N.hispanica*	**ML8**	*Lumaapiculata*	**Italy**	**PP146596**	**PP174985**	**PP197173**
*N.hispanica*	**MP18**	*Myrtuscommunis* ‘Microphylla’	**Italy**	**PP146599**	**PP174986**	**PP197174**
*N.hispanica*	**MP20**	*Myrtuscommunis* ‘Microphylla’	**Italy**	**PP146600**	**PP174987**	**PP197175**
*N.hispanica*	**MT38**	Myrtuscommunissubsp.tarentina	**Italy**	**PP146606**	**PP174989**	**PP197176**
*N.hispanica*	**MT39**	Myrtuscommunissubsp.tarentina	**Italy**	**PP146607**	**PP174990**	**PP197177**
*N.honoluluana*	CBS 114495^T^	*Telopea* sp.	USA	KM199364	KM199548	KM199457
*N.hydeana*	MFLUCC 20-0132^T^	*Artocarpusheterophyllus*	Thailand	MW266069	MW251129	MW251119
*N.hyperici*	KUNCC 22-12597^T^	*Hypericummonogynum*	China	OP498010	OP713768	OP765908
*N.iberica*	CBS 147688^T^	*Eucalyptusglobulus*	Portugal	MW794111	MW805402	MW802844
*N.iberica*	CBS 147689	*Eucalyptusglobulus*	Spain	MW794114	MW805403	MW802847
*N.iberica*	CSUFTCC91	*Camelliaoleifera*	China	OK493587	OK507957	OK562362
*N.iberica*	CSUFTCC92	*Camelliaoleifera*	China	OK493588	OK507958	OK562363
*N.iberica*	CSUFTCC93	*Camelliaoleifera*	China	OK493589	OK507959	OK562364
*N.iberica*	**MP13**	*Myrtuscommunis* ‘Microphylla’	**Italy**	**PP146587**	**PP174967**	**PP197167**
*N.iranensis*	CBS 137768^T^	Fragaria×ananassa	Iran	KM074048	KM074051	KM074057
*N.javaensis*	CBS 257.31^T^	*Cocosnucifera*	Indonesia	KM199357	KM199548	KM199457
*N.keteleeriae*	MFLUCC 13-0915^T^	*Keteleeriapubescens*	China	KJ023087	KJ023089	KJ023088
*N.longiappendiculata*	CBS 147690^T^	*Eucalyptusglobulus*	Portugal	MW794112	MW805404	MW802845
*N.lusitanica*	CBS 147692^T^	*Eucalyptusglobulus*	Portugal	MW794110	MW805406	MW802843
*N.macadamiae*	BRIP 63737c^T^	*Macadamiaintegrifolia*	Australia	KX186604	KX186627	KX186654
*N.maddoxii*	BRIP 72266a^T^	*Macadamiaintegrifolia*	Australia	MZ303782	MZ344167	MZ312675
*N.magna*	MFLUCC 12-0652^T^	*Pteridium* sp.	France	KF582795	KF582791	KF582793
*N.mesopotamica*	CBS 336.86^T^	*Pinusbrutia*	Turkey	KM199362	KM199555	KM199441
*N.mianyangensis*	CGMCC 3.23554^T^	*Paeoniasuffruticosa*	China	OP546681	OP723490	OP672161
*N.musae*	MFLUCC 15-0776^T^	*Musa* sp.	Thailand	NR_156311	KX789685	KX789686
*N.natalensis*	CBS 138.41^T^	*Acaciamollissima*	South Africa	NR_156288	KM199552	KM199466
*N.nebuloides*	BRIP 66617^T^	*Sporobolusjacquemontii*	Australia	MK966338	MK977633	MK977632
*N.olumideae*	BRIP 72273a^T^	*Macadamiaintegrifolia*	Australia	MZ303790	MZ344175	MZ312683
*N.paeoniae-suffruticosae*	CGMCC 3.23555^T^	*Paeoniasuffruticosa*	China	OP082292	OP204794	OP235980
*N.pandanicola*	KUMCC 17-0175^T^	*Pandanus* sp.	China	N/A	MH388389	MH412720
*N.pernambucana*	URM 7148-01^T^	*Vismiaguianensis*	Brazil	KJ792466	KU306739	N/A
*N.perukae*	FMBCC 11.3^T^	*Psidiumguajava*	Pakistan	MH209077	MH523647	MH460876
*N.petila*	MFLUCC 17-1738^T^	*Rhizophoraapiculata*	Thailand	MK764276	MK764320	MK764342
*N.phangngaensis*	MFLUCC 18-0119^T^	*Pandanus* sp.	Thailand	MH388354	MH388390	MH412721
*N.photiniae*	MFLUCC 22-0129^T^	*Photiniaserratifolia*	China	OP498008	OP753368	OP752131
*N.piceana*	CBS 394.48^T^	*Picea* sp.	UK	KM199368	KM199527	KM199453
*N.protearum*	CBS 114178^T^	*Leucospermumcuneiforme*	Zimbabwe	JN712498	KM199542	KM199463
*N.psidii*	FMBCC 11.2^T^	*Psidiumguajava*	Pakistan	MF783082	MH460874	MH477870
*N.rhapidis*	GUCC 21501^T^	*Rhododendronsimsii*	China	MW931620	MW980442	MW980441
*N.rhizophorae*	MFLUCC 17-1551^T^	*Rhizophoramucronata*	Thailand	MK764277	MK764321	MK764343
*N.rhododendri*	GUCC 21504^T^	*Rhododendronsimsii*	China	MW979577	MW980444	MW980443
*N.rhododendricola*	KUNCC 22-10802^T^	*Rhododendron* sp.	China	OK283069	OK274148	OK274147
*N.rosae*	CBS 101057^T^	*Rosa* sp.	New Zealand	KM199359	KM199523	KM199429
*N.rosae*	CBS 124745	*Paeoniasuffruticosa*	USA	KM199360	KM199524	KM199430
*N.rosae*	CRM-FRC	Fragaria×ananassa	Mexico	MN385718	MN268532	MN268529
*N.rosae*	AC50	*Perseaamericana*	Italy	ON117810	ON107276	ON209165
*N.rosae*	**CV46**	*Callistemonviminalis*	**Italy**	**PP146591**	**PP174971**	**PP197179**
*N.rosae*	**CV47**	*Callistemonviminalis*	**Italy**	**PP146592**	**PP174972**	**PP197180**
*N.rosae*	**CV51**	*Callistemonviminalis*	**Italy**	**PP146584**	**PP174982**	**PP197191**
*N.rosae*	**ML1**	*Lumaapiculata*	**Italy**	**PP146583**	**PP174973**	**PP197181**
*N.rosae*	**ML6**	*Lumaapiculata*	**Italy**	**PP146595**	**PP174974**	**PP197182**
*N.rosae*	**MP10**	*Myrtuscommunis* ‘Microphylla’	**Italy**	**PP146597**	**PP174975**	**PP197183**
*N.rosae*	**MP15**	*Myrtuscommunis* ‘Microphylla’	**Italy**	**PP146598**	**PP174976**	**PP197184**
*N.rosae*	**MP21**	*Myrtuscommunis* ‘Microphylla’	**Italy**	**PP146601**	**PP174977**	**PP197185**
*N.rosae*	**MP25**	*Myrtuscommunis* ‘Microphylla’	**Italy**	**PP146588**	**PP174988**	**PP197186**
*N.rosae*	**MP28**	*Myrtuscommunis* ‘Microphylla’	**Italy**	**PP146602**	**PP174978**	**PP197187**
*N.rosae*	**MT32**	Myrtuscommunissubsp.tarentina	**Italy**	**PP146603**	**PP174979**	**PP197188**
*N.rosae*	**MT35**	Myrtuscommunissubsp.tarentina	**Italy**	**PP146604**	**PP174980**	**PP197189**
*N.rosae*	**MT37**	Myrtuscommunissubsp.tarentina	**Italy**	**PP146605**	**PP174981**	**PP197190**
*N.rosicola*	CFCC 51992^T^	*Rosachinensis*	China	KY885239	KY885243	KY885245
*N.rosicola*	**MP29**	*Myrtuscommunis* ‘Microphylla’	**Italy**	**PP146590**	**PP174968**	**PP197168**
*N.samarangensis*	MFLUCC 12-0233^T^	*Syzygiumsamarangense*	Thailand	JQ968609	JQ968611	JQ968610
*N.saprophytica*	MFLUCC 12-0282^T^	*Magnolia* sp.	China	JX398982	JX399048	JX399017
*N.scalabiensis*	CAA1029^T^	*Vacciniumcorymbosum*	Portugal	MW969748	MW959100	MW934611
*N.sichuanensis*	CFCC 54338^T^	*Castaneamollissima*	China	MW166231	MW199750	MW218524
*N.siciliana*	CBS 149117	*Perseaamericana*	Italy	ON117813	ON107273	ON209162
*N.sonneratiae*	MFLUCC 17-1745^T^	*Sonneronataalba*	Thailand	MK764280	MK764324	MK764346
*N.steyaertii*	IMI 192475^T^	*Eucalytpusviminalis*	Australia	KF582796	KF582792	KF582794
*N.subepidermalis*	CFCC 55160^T^	*Rosarugosa*	China	OK560699	OM622425	OM117690
*N.suphanburiensis*	MFLUCC 22-0126^T^	dead stem of unid. plant	Thailand	OP497994	OP753372	OP752135
*N.surinamensis*	CBS 450.74^T^	Soil under *Elaeisguineensis*	Suriname	KM199351	KM199518	KM199465
*N.terricola*	CGMCC 3.23553^T^	*Paeoniasuffruticosa*	China	OP082294	OP204796	OP235982
*N.thailandica*	MFLUCC 17-1730^T^	*Rhizophoramucronata*	Thailand	MK764281	MK764325	MK764347
*N.umbrinospora*	MFLUCC 12-0285^T^	unidentified plant	China	JX398984	JX399050	JX399019
*N.vaccinii*	CAA1059^T^	*Vacciniumcorymbosum*	Portugal	MW969747	MW959099	MW934610
*N.vaccinii*	22-Jan	*Vacciniumcorymbosum*	Serbia	OQ316613	OQ342778	OQ473026
*N.vaccinii*	21-Feb	*Vacciniumcorymbosum*	Serbia	OQ316612	OQ342777	OQ473025
*N.vaccinii*	19-Jul	*Vacciniumcorymbosum*	Serbia	OQ316611	OQ342776	OQ473024
*N.vacciniicola*	CAA1055^T^	*Vacciniumcorymbosum*	Portugal	MW969751	MW959103	MW934614
*N.vheenae*	BRIP 72293a^T^	*Macadamiaintegrifolia*	Australia	MZ303792	MZ344177	MZ312685
*N.vitis*	MFLUCC 15-1265^T^	*Vitisvinifera*	China	KU140694	KU140676	KU140685
*N.xishuangbannaensis*	KUMCC 21-0424^T^	*Kerivoulahardwickii*	China	ON426865	OR025973	OR025934
*N.zakeelii*	BRIP 72282a^T^	*Macadamiaintegrifolia*	Australia	MZ303789	MZ344174	MZ312682
*N.zakeelii*	BRIP 72271°	*Macadamiaintegrifolia*	Australia	MZ303788	MZ344173	MZ312681
*N.zakeelii*	**CV52**	*Callistemonviminalis*	**Italy**	**PP146585**	**PP174969**	**PP197170**
*N.zakeelii*	**MP30**	*Myrtuscommunis* ‘Microphylla’	**Italy**	**PP146589**	**PP174970**	**PP197169**
*N.zimbabwana*	CBS 111495^T^	*Leucospermumcuneiforme*	Zimbabwe	JX556231	KM199545	KM199456
*N.zingiberis*	GUCC 21001^T^	*Zingiberofficinale*	China	MW930715	MZ683389	MZ683390
*Pestalotiopsisbiciliata*	CBS 124463^T^	Platanus×hispanica	Slovakia	KM199308	KM199505	KM199399
*P.biciliata*	CBS 790.68	*Taxusbaccata*	Netherlands	KM199305	KM199507	KM199400
*P.biciliata*	**MP11**	*Myrtuscommunis* ‘Microphylla’	**Italy**	**PP146582**	**PP174966**	**PP197166**
*P.brachiata*	LC2988^T^	*Camellia* sp.	China	KX894933	KX895150	KX895265
*P.colombiensis*	CBS 118553^T^	Eucalyptusgrandis×urophylla	Colombia	KM199307	KM199488	KM199421
*P.diversiseta*	MFLUCC 12-0287^T^	Dead plant material	China	NR_120187	JX399073	JX399040

These results were also confirmed by the nucleotide comparisons (Table 3), which revealed (almost) identical sequences to the ex-type strains of the respective species. While within *N.hispanica* all markers of all isolates were identical with the ex-type sequences, within *N.rosae* slight sequence differences were observed in three isolates, viz. ML1 (1 nucleotide substitution and 1 gap in ITS), CV51 and MP25 (1 gap in ITS and 1 nucleotide substitution in *tef1*). Within *N.camelliae-oleiferae*, the ITS differed from the ex-type sequence by two gaps; however, these are likely sequencing errors as they were located in polyA regions at the immediate beginning of the deposited sequence which do not sufficiently resolved by the Sanger sequencing method. In *N.zakelii*, the ITS and *tub2* sequences of our isolates differed by 3 nucleotide substitutions from the ex-type sequences; however, the difference to the second verified strain (BRIP 72271a) was much less (1 substitution in ITS, identical *tef1* and *tub2* sequences). Also, in *P.biciliata* the ITS differed by 3 and 1 nucleotide substitutions in ITS and *tef1*, respectively, but only by 1 nucleotide substitution in *tef1* from the second verified strain (CBS 790.68). The species identification of these isolates with some differences to the ex-type sequences was also confirmed by BLAST searches of *tef1* and *tub2*, in which the respective species were revealed as closest matches with 100%.

**Table 3.** Sequence comparison of differences in number of nucleotide substitutions and gaps between the sequences of isolates of the present study and the ex-type sequences of the respective species.

Species	No. of isolates	Sequence difference to ex-type strain (nucleotide substitutions/gaps)		
		ITS	*tef1*	*tub2*
*Neopestalotiopsiscamelliae-oleiferae*	1	(2)*/0	0/0	0/0
*N.hispanica* (syn. *N.vaccinii*)	8	0/0	0/0	0/0
*N.iberica*	1	0/0	1/0	0/0
*N.rosae*	13	0-1/0-1	0-1/0	0/0
*N.rosicola*	1	0/0	1/0	0/0
*N.zakeelii*	2	3/0	0/0	2/0
*Pestalotiopsisbiciliata*	1	3/0	1/1	0/0

*Gaps located at the immediate beginning of the ITS in polyA regions, likely representing sequencing errors.

Morphology of the conidia (conidial shape, size of the conidial body, number and coloration of the conidial cells, number and length of appendages) matched the genera and species and was therefore in line with the molecular identification; however, as within the genus *Neopestalotiopsis* conidial shapes and sizes largely overlap and are highly similar in the bulk of these cryptic species, no species identification is possible based on morphology.

### ﻿Pathogenicity test

**Figure 3.**
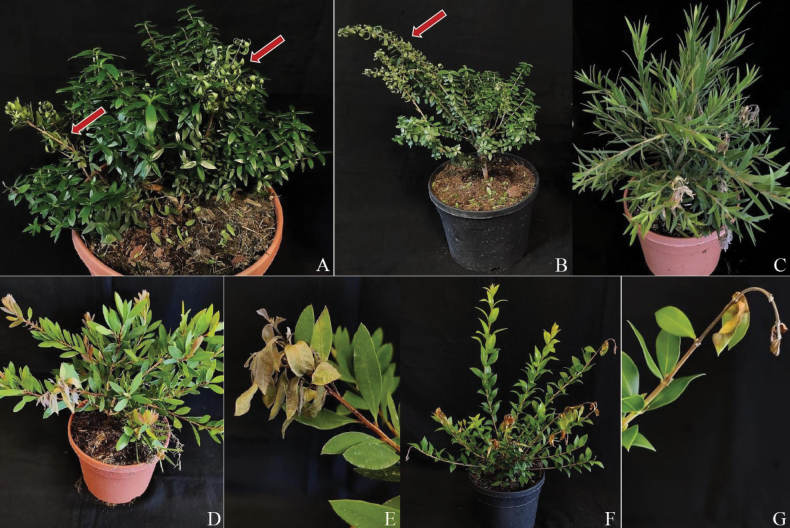
Symptoms caused by artificial inoculation with *Neopestalotiopsisrosae* MT32 on *Myrtuscommunis* ‘Microphylla’ **A**Myrtuscommunissubsp.tarentina (red arrow) **B***Callistemonviminalis* (red arrow) **C***Callistemonlaevis***D–E***Lumaapiculata***F–G**.

The results of the pathogenicity tests (disease incidence) are shown in Table 4. Although some cankers were visible after one week, the size of the twigs was too thin and small to assess the disease severity (lesion length). Only *N.rosae* and *N.hispanica* caused disease symptoms on all hosts tested, whereas *P.biciliata* did not show pathogenicity. Moreover, the isolates inoculated on wounded twigs caused similar symptoms on different host species and showed twig canker and dieback (Fig. 3).

**Table 4.** Results of pathogenicity tests conducted in this study.

Host	*Neopestalotiopsis* spp. isolates						
	***N.rosae* MT32**	***N.rosicola* MP29**	***N.hispanica* ML8**	***N.iberica* MP13**	***N.zakeelii* MP30**	***N.camelliae-oleiferae* ML3**	***P.biciliata* MP11**
*Callistemonlaevis*	+	+	+	+	+	+	-
*C.viminalis*	+	+	+	+	+	+	-
*Lumaapiculata*	+	-	+	-	-	-	-
*Myrtuscommunis* ‘Microphylla’	+	+	+	+	+	+	-
M.communissubsp.tarentina	+	+	+	+	-	+	-

“+” symptoms; “–” symptomless.

## ﻿Discussion

In the present study, the following fungal species were described causing twig canker, dieback and defoliation on *Callistemon*, *Luma* and *Myrtus* spp.: *Neopestalotiopsiscamelliae-oleiferae*, *N.hispanica* (syn. *N.vaccinii*), *N.iberica*, *N.rosae*, *N.rosicola*, *N.zakeelii*, and *Pestalotiopsisbiciliata*. In the group of fungi characterized in this study the species *N.hispanica* and *N.rosae*, based on frequency of isolations and ability to induce symptoms in all tested plant species, probably play a key role in the disease development. *Neopestalotiopsis* species, as well as other species belonging to other taxa within the Sporocadaceae are known worldwide causing different symptoms on many hosts, such as leaf spot, flower and fruit blight, twig canker and crown rot (Rebollar-Alviter et al. 2020; Santos et al. 2022; Shahriar et al. 2022; Guterres et al. 2023). Similar symptoms to those observed in this study have been reported for *N.iberica* on *Eucalyptusglobulus* in Portugal (Diogo et al. 2021) and on *Synsepalumdulcificum* in China (You et al. 2024), for *N.rosae* on blueberry in Peru, and Portugal (Rodríguez-Gálvez et al. 2020; Santos et al. 2022), and on avocado in Italy (Fiorenza et al. 2022b), for *N.rosicola* on *Rosachinensis* in China (Jiang et al. 2018), for *N.hispanica* (syn. *N.vaccinii*) on *E.globulus* in Portugal and Spain (Diogo et al. 2021) and on blueberry in Portugal (Santos et al. 2022). However, *N.camelliae-oleiferae* was also reported causing foliar symptoms on *Camelliaoleifera* in China (Li et al. 2021), and *N.zakeelii* was described in 2021, associated with flower diseases of *Macadamiaintegrifolia* in Australia (Prasannath et al. 2021). Although *N.zakeelii* was reported from macadamia inflorescences showing dry flowers, Koch’s postulates were not conducted and the pathogenicity was not demonstrated (Prasannath et al. 2021). In our study it is the first time that *N.zakeelii* is reported to be associated with symptoms of canker and dieback and our study confirms, for the first time, the pathogenic activity on *C.laevis* and *viminalis* and on *M.communis* ‘Microphylla’ causing necrotic lesions of woody tissues. On the other hand, different results have been obtained regarding pathogenicity of *P.biciliata*. This species was reported around the world causing also twig canker and dieback on different hosts such as blueberry, *Pinuspinea*, *Quercuscoccifera* and *Pistacialentiscus* (Hlaiem et al. 2022 a, b; Santos et al. 2022), whereas in our study, although isolated from cankered tissues, the tested isolate did not show any pathogenic activity on these hosts. This result is not surprising, since many other studies demonstrated that pestalotioid fungi can occur as saprobionts or generalist endophytes (Wu et al. 1982; Agarwal and Chauhan 1988; Yanna and Hyde 2002; Osono and Takeda 2006; Hu et al. 2007; Rajulu et al. 2014; Prakash et al. 2015; Park et al. 2016; Reddy et al. 2016). The results of our study showed the potential fungal diversity involved in twig canker. In fact, as highlighted in this study, to investigate these kinds of diseases, such as canker and dieback, the whole fungal diversity and the interactions among all the species involved must be taken into consideration.

## ﻿Additional information

### Author ORCIDs

Dalia Aiello https://orcid.org/0000-0002-6018-6850

Giorgio Gusella https://orcid.org/0000-0002-0519-1200

Giuseppa Rosaria Leonardi https://orcid.org/0000-0002-4676-5100

Giancarlo Polizzi https://orcid.org/0000-0001-8630-2760

Hermann Voglmayr https://orcid.org/0000-0001-7666-993X

### Data availability

All of the data that support the findings of this study are available in the main text
